# Photosynthesis-Inhibiting Activity of 1-[(2-Chlorophenyl)carbamoyl]- and 1-[(2-Nitrophenyl)carbamoyl]naphthalen-2-yl Alkylcarbamates †

**DOI:** 10.3390/molecules22071199

**Published:** 2017-07-17

**Authors:** Tomas Gonec, Josef Stranik, Matus Pesko, Jiri Kos, Michal Oravec, Katarina Kralova, Josef Jampilek

**Affiliations:** 1 Department of Chemical Drugs, Faculty of Pharmacy, University of Veterinary and Pharmaceutical Sciences, Palackeho 1, 61242 Brno, Czech Republic; stranik27@seznam.cz; 2 Department of Environmental Ecology, Faculty of Natural Sciences, Comenius University, Ilkovicova 6, 84215 Bratislava, Slovakia; matus.pesko@gmail.com; 3 Department of Pharmaceutical Chemistry, Faculty of Pharmacy, Comenius University, Odbojarov 10, 83232 Bratislava, Slovakia; jurd@email.cz; 4 Global Change Research Institute CAS, Belidla 986/4a, 60300 Brno, Czech Republic; oravec.m@czechglobe.cz; 5 Institute of Chemistry, Faculty of Natural Sciences, Comenius University, Ilkovicova 6, 84215 Bratislava, Slovakia; kata.kralova@gmail.com

**Keywords:** alkylcarbamates, hydroxynaphthalene-carboxamides, PET inhibition, spinach chloroplasts, structure-activity relationships

## Abstract

Eight 1-[(2-chlorophenyl)carbamoyl]naphthalen-2-yl alkylcarbamates and eight 1-[(2-nitrophenyl)carbamoyl]naphthalen-2-yl alkylcarbamates were tested for their activity related to the inhibition of photosynthetic electron transport (PET) in spinach (*Spinacia oleracea* L.) chloroplasts. The PET-inhibiting activity of the compounds was relatively low; the corresponding IC_50_ values ranged from 0.05 to 0.664 mmol/L; and the highest activity within the series of compounds was observed for 1-[(2-chlorophenyl)-carbamoyl]naphthalen-2-yl propylcarbamate. It has been proven that the compounds are PET-inhibitors in photosystem II. Despite rather low PET-inhibiting activities, primary structure-activity trends can be discussed.

## 1. Introduction

Although naphthalene can be considered as the simplest compound from the group of arenes, it is one of the most interesting arenes. Naphthalene-based drugs include not only clinically used anti-infective chemotherapeutics—e.g., naftifine, terbinafine, tolnaftate, nafcillin—but also other compounds with significant antimicrobial effects, e.g., dye naftol [1,2,3]. The naphthalene scaffold can be found in many other bioactive compounds [1,3,4,5,6,7,8]; therefore, this scaffold can be considered a privileged structure [9,10,11,12].

Our research group prepared and tested naphthalenecarboxamides and various positional isomers of hydroxynaphthalenecarboxamides as potential antimicrobial and antiprotozoal compounds [13,14,15,16,17,18,19,20,21,22]. The presence of an amide (–CONH–) and/or a carbamate (–OCONH–) group(s) in the structure of compounds enables interactions with various enzymes or enzymatic systems ([23,24,25,26] and references therein). In addition, these moieties can be found in many herbicides acting as photosynthesis inhibitors, e.g., [27,28,29,30,31,32,33,34,35]. Though currently about 20 mechanisms of action of herbicides are known [36], over 50% of marketed herbicides act by reversible binding to photosystem II (PS II) [37], resulting in interruption of the photosynthetic electron transport (PET) [38,39,40]. Various types of substituents modify properties of amide and carbamate moieties [41,42].

In the middle of the 1970s, it was found that salicylanilides belong to effective uncoupling agents of oxidative phosphorylation [43,44,45], and acceleration of the deactivation reactions of water splitting enzyme system Y by 3-*tert*-butyl-5-chloro-*N*-(2-chloro-4-nitrophenyl)-2-hydroxybenzamide was observed [44]. Substituted salicylanilides or their bioisosteres inhibited PET in spinach chloroplasts [13,14,15,16,17,18,31,32,33,34,35] and reduced chlorophyll content in green alga, *Chlorella vulgaris* [31,35,46,47]. It is important to note that in addition to the above-mentioned herbicidal activity, the wide spectrum of biological effects of salicylanilides includes, for example, antibacterial, antimycobacterial, antifungal, and anthelmintic activity; however, their mechanism of action is still under investigation ([25,26] and references therein).

In the context of the above-mentioned facts, 1-[(2-chlorophenyl)-carbamoyl]naphthalen-2-yl alkylcarbamates and 1-[(2-nitrophenyl)carbamoyl]naphthalen-2-yl alkylcarbamates were prepared [22] and tested for their photosynthesis-inhibiting activity—the PET inhibition in spinach chloroplasts (*Spinacia oleracea* L.). The structure–activity relationships are discussed.

## 2. Results and Discussion

### 2.1. Chemistry

**Scheme 1:**
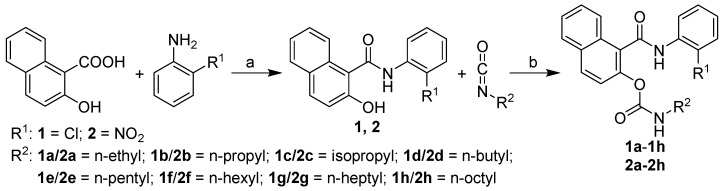
Synthesis of 1-[(2-chlorophenyl)carbamoyl]naphthalen-2-yl carbamates **1a**–**1h** and 1-[(2-nitrophenyl)carbamoyl]naphthalen-2-yl carbamates **2a**–**2h** [22]. Reagents and conditions: (a) PCl_3_, chlorobenzene, MW; (b) TEA, acetonitrile, room temperature.

A microwave-assisted synthesis [15] gave *N*-(2-chlorophenyl)-2-hydroxynaphthalene-1-carboxamide (**1**) and *N*-(2-nitrophenyl)-2-hydroxynaphthalene-1-carboxamide (**2**). Then these pattern compounds **1** and **2** with triethylamine and appropriate alkyl isocyanates yielded a series of eight 1-[(2-chlorophenyl)carbamoyl]naphthalen-2-yl carbamates **1a**–**1h** and eight 1-[(2-nitrophenyl)-carbamoyl]naphthalen-2-yl carbamates **2a**–**2h**, see Scheme 1 [22].

### 2.2. Inhibition of Photosynthetic Electron Transport (PET) in Spinach Chloroplasts

The PET-inhibiting activity was expressed by IC_50_ value (compound concentration in mol/L causing 50% inhibition of PET), see Table 1. Both pattern anilides **1** and **2** showed higher PET-inhibiting activity than their carbamate counterparts. The highest activity within the series of the chlorinated carbamates **1a**–**1h** (series *I*) was observed for 1-[(2-chlorophenyl)carbamoyl]-naphthalen-2-yl propylcarbamate (**1b**, IC_50_ = 0.08 mM), while the highest PET-inhibiting activity within the series of the nitrated carbamates **2a**–**2h** (series *II*) was observed for 1-[(2-nitrophenyl)-carbamoyl]naphthalen-2-yl pentylcarbamate (**2e**, IC_50_ = 0.233 mM), Table 1. Despite rather low PET-inhibiting activities, primary dependences between structure of the compounds and their PET inhibition can be discussed.

ACD/Percepta ver. 2012 was used for prediction of various physicochemical descriptors, from which only those that best characterize the influence of PET-inhibiting activity on compound structure are listed in Table 1. The lipophilicity of compounds **1a**–**1h**, expressed as calculated log *P* (clogP) values, ranged from 3.94 (compound **1a**, R = C_2_H_5_) to 7.19 (compound **1h**, R = C_8_H_17_), while the clogP values of compounds **2a**–**2h** ranged from 3.58 (compound **2a**, R = C_2_H_5_) to 7.22 (compound **2h**, R = C_8_H_17_). Lipophilicity increases with the lengthening of the alkyl tail. Propyl showed a higher clogP value than isopropyl. In general, it can be stated that lipophilicity of these compounds is rather high. Recommended log *P* value for drugs and agrochemicals is ≤5 [48]. The bulkiness of individual substituents R^2^ expressed as molar volume MV [cm^−3^] was calculated also for the hydrophobic *N*-alkyl tail; its values ranged from 47.29 to 146.33. This parameter represents the bulk of substituents (i.e., tail length/branching) of each compound relative to other members of the same series. Taft polar constants σ* representing electronic properties of individual alkyl substituents of the discussed compounds were also included in Table 1; they ranged from −0.25 to −0.11.

**Figure 1:**
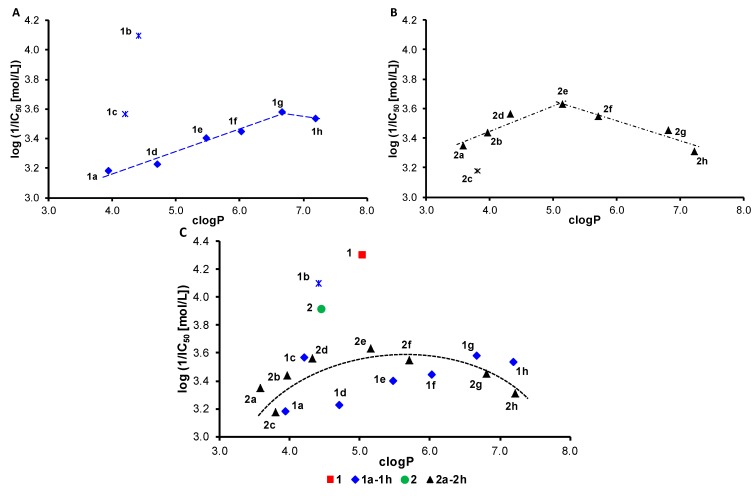
Dependence of PET-inhibiting activity log(1/IC_50_ [mol/L]) of all discussed compounds **1a**–**1h** (**A**), **2a**–**2h** (**B**) and **1**–**2h** (**C**) in spinach chloroplasts on lipophilicity expressed as clogP.

The dependence of the PET-inhibiting activity expressed as log(1/IC_50_ [mol/L]) of compounds **1**, **1a**–**1h** and **2**, **2a**–**2h** in spinach chloroplasts on lipophilicity expressed as clogP is shown in Figure 1A,B, while Figure 1C illustrates this dependence for all investigated compounds **1**–**2h**.

Anilide **1** of series *I* was considerably more active than *N*-alkyl substituted compounds **1a**–**1h**. While ethyl derivative **1a** of series *I* was inactive due to low lipophilicity, propyl derivative **1c**—showing sufficient lipophilicity together with suitable aqueous solubility—was the most active compound. With the elongation of the alkyl chain in the R substituent, the aqueous solubility of the evaluated derivatives decreased, and at higher concentrations they precipitated from the solution during the experiment. Among compounds of series *I*, the lowest solubility was shown by butyl derivative **1d**, and the solubility of derivatives **1d**–**1h** with longer alkyl chains was similar and significantly lower than that of propyl **1b** and isopropyl **1c** derivatives, which resulted in a notable activity decrease (Figure 1A). A slight increase of PET-inhibiting activity with further prolongation of the alkyl tail can be connected with the fact that a longer alkyl chain can be incorporated in the thylakoid membrane to a greater extent and subsequently cause membrane perturbation also at lower concentrations. The dependences of the PET-inhibiting activity log(1/IC_50_ [mol/L]) of compounds **2a**–**2h** on clogP was bilinear, pentyl derivative **2e** being the most effective PET inhibitor (Figure 1B). The lower activity of isopropyl derivative **2c** could be connected with its lower aqueous solubility. The dependence of log(1/IC_50_ [mol/L]) on clogP for all the investigated compounds is illustrated in Figure 1C. It is evident that with the exception of compounds **1b** and **1c** of series *I* for compounds with clogP < 6.57 the activity of compounds of series *II* was slightly higher than that of compounds of series *I* with comparable lipophilicity. Lower PET-inhibiting activity of heptyl **2g** and octyl **2h** derivatives of series *II* compared to their analogues **1g**, **1h** of series *I* could be connected with their more significant solubility decrease with the elongation of the alkyl chain in the R^2^ substituent, resulting in precipitation from the solution during the experiment.

**Figure 2:**
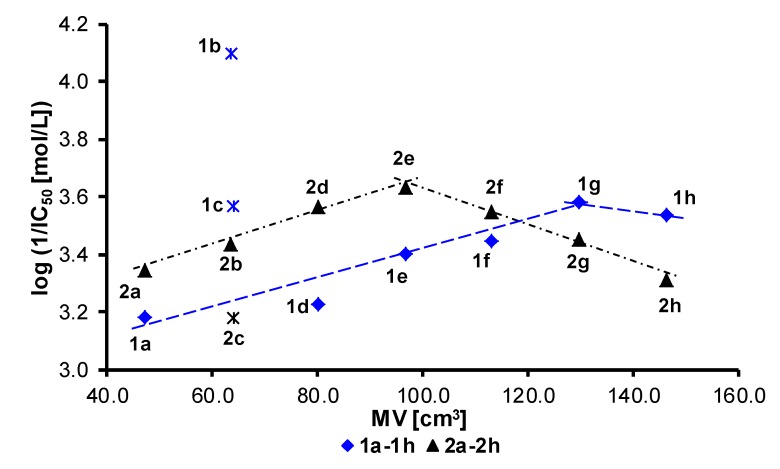
Dependence of PET-inhibiting activity log(1/IC_50_ [mol/L]) of carbamates **1a**–**1h** and **2a**–**2h** in spinach chloroplasts on bulkiness of R^2^ substituents expressed as molar volume MV [cm^−3^] of alkyl tail of compounds.

After exclusion of compounds **1a**, **1b**, and **2c**, a bilinear course was found also for the dependences of the PET-inhibiting activity on log(1/IC_50_ [mol/L]) of cabamate series *I* and *II* in spinach chloroplasts on bulkiness expressed as molar volume MV of the alkyl tails R^2^, see Figure 2. The PET-inhibiting activity within the nitrated series *II* linearly increased with the increase of molar volume (influence of substituent R bulkiness, r = 0.9949, *n* = 4) up to pentyl derivative **2e** (MV = 96.81 cm^3^). After this optimum, activity showed a strong linear decrease with the subsequent increase of molar volume up to MV = 146.33 cm^3^ (**2h**, r = −0.9923, *n* = 4). On the other hand, PET inhibition within the chlorinated series showed a moderate linear increase with the increase of molar volume (r = 0.9577, *n* = 5) up to heptyl derivative **1g** (MV = 129.83 cm^3^) and, after that, slightly decreased to octyl derivative **1h** (MV = 146.33 cm^3^).

It is important to note that a strong dependence of PET inhibition on the electron-withdrawing effect of substituents in individual series of many PET inhibitors was observed [14,15,16,34,49]. Therefore, it can be hypothesized that also a nitro moiety in the *ortho* position of the anilide ring (electronic Hammett’s parameter σ = 1.72 [50]) activates more strongly an amide bond—one of the structural motifs responsible for binding to PS II—and from this point of view, it is more advantageous than chlorine in the *ortho* position (electronic Hammett’s parameter σ = 0.67 [50]) of the anilide ring. In general, the *N*-alkyl tail of a suitable length facilitates penetration of a compound through hydrophobic regions of thylakoid membrane to the site of action in photosynthetic apparatus, as discussed below, but the electron-deficient amide bond is more important for the intrinsic effect of compounds [14,15,16,34,35,51]. Therefore, it is noteworthy that the PET-inhibiting activity of pentyl derivative **2e** (IC_50_ = 0.233 mM, MV = 96.81 cm^3^) is similar to the PET inhibition of heptyl derivative **1g** (IC_50_ = 0.263 mM, MV = 129.83 cm^3^) although MV value is significantly lower for compound **2e**.

**Figure 3:**
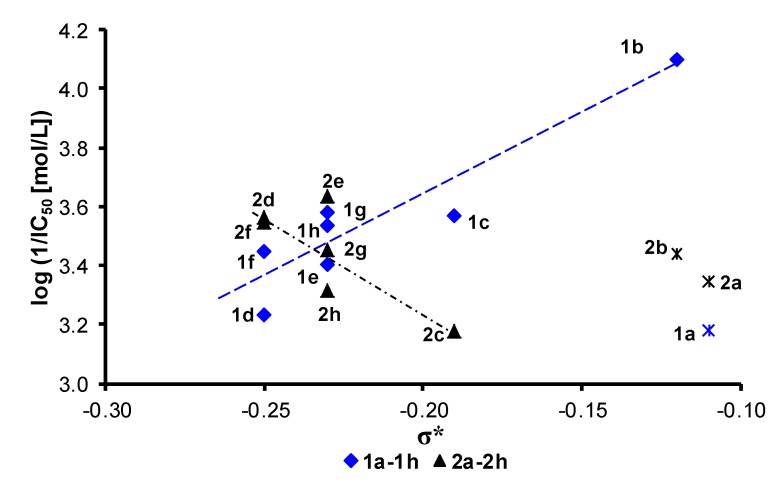
Dependence of PET-inhibiting activity log (1/IC_50_ [mol/L]) of studied carbamates **1a**–**1h** and **2a**–**2h** on electronic properties expressed as Taft polar constants σ* of alkyl tail R^2^.

The dependence of PET-inhibiting activity of studied compounds **1a**–**1h** and **2a**–**2h** on Taft polar constants σ* of the alkyl tail R^2^ is shown in Figure 3. With the exception of compounds with the highest σ* values in both series belonging to compounds with short alkyl chains (ethyl **1a** as well as ethyl **2a** and propyl **2b** derivatives), the observed trend for the two studied series was opposite. While for compounds of series *I*, the increasing σ* value resulted in increased inhibitory activity, for compounds of series *II* it showed a decrease. Therefore, it can be hypothesized that these different properties/behaviour of compounds of series *I* and *II*, as mentioned above, are caused by possible interactions and the electron activation of amide and carbamate groups (responsible also for interactions with the photosynthetic apparatus) with the spatially close NO_2_ moiety in the *ortho* position of the anilide ring.

Besides physicochemical parameters—for example, lipophilicity or electronic properties of substituents—an appropriate concentration of the compound at the site of action in the photosynthetic apparatus is also important for PET-inhibiting activity. A compound having very low aqueous solubility cannot pass through the hydrophilic regions of the thylakoid membrane to reach the site of action, which results in a significant decrease of inhibitory activity. The solubility of butyl derivative **1d** and derivatives with longer alkyl chains was similar and significantly lower than that of propyl **1b** and isopropyl **1c** derivatives, which resulted in a notable activity decrease; a slight increase of PET-inhibiting activity with a further prolongation of the alkyl tail can be connected with the fact that a longer alkyl chain can be incorporated in the thylakoid membrane to a greater extent and subsequently cause membrane perturbation also at a lower concentration. This effect is connected with the surface activity of these compounds (they can be considered as non-ionic surfactants) and with the alkyl tail length (molar volume), which is again reflected by lipophilicity. From the aspect of PET-inhibiting activity, the lipophilicity optimum for C_4_–C_8_ alkyl chains can be found at C_7_ (compound **1g**) and C_5_ (compound **2e**), see Figure 1 and Figure 2. With the further elongation of the alkyl chain (hydrophobic part) to octyl, so called ‘cut-off’ effect—i.e., the loss/notable decrease of biological activity usually observed for amphiphilic compounds—was manifested [26,27,52,53,54].

The application of 2,5-diphenylcarbazide (DPC, artificial electron donor) that supplies electrons in the site of Z^•^/D^•^ intermediate, i.e., tyrosine radicals Tyr_Z_ and Tyr_D_ (or their surroundings) that are situated in D_1_ and D_2_ proteins on the donor side of PS II [40] in chloroplasts, the activity of which was inhibited by the most active compounds **1b** or **2e** (up to 30% of the control), caused practically complete PET restoration already at the addition of three-fold DPC concentration with regard to the applied concentration of compound **2e**. Therefore, it can be concluded that the site of action of studied alkylcarbamates, **1a**–**1h** and **2a**–**2h**, is situated mainly on the donor side of PS II. The site of action situated on the donor side of PS II was found also for 2-alkylthio-6-R-benzothiazoles (R = 6-formamido-, 6-acetamido-, and 6-benzoylamino-) [55], anilides of 2-alkylpyridine-4-carboxylic acids [56], cationic surfactants [57,58] acting in the intermediates Z^•^/D^•^ and 2-alkylsulphanyl-4-pyridinecarbothioamides acting in the D^•^ intermediate [59].

## 3. Experimental Section

### 3.1. Synthesis

Both pattern compounds *N*-(2-chlorophenyl)-2-hydroxynaphthalene-1-carboxamide (**1**) and *N*-(2-nitrophenyl)-2-hydroxynaphthalene-1-carboxamide (**2**) as well as all carbamates **1a**–**1h** and **2a**–**2h** were described recently by Gonec et al. [15,22].

### 3.2. Study of Photosynthetic Electron Transport (PET) Inhibition in Spinach Chloroplasts

Chloroplasts were prepared from spinach (*Spinacia oleracea* L.) according to Masarovicova and Kralova [60]. The PET inhibition in isolated spinach chloroplasts was performed as described recently [15] using the artificial electron acceptor 2,6-dichlorophenol-indophenol (DCPIP). The rate of photosynthetic electron transport was monitored as a photoreduction of DCPIP. The inhibitory efficiency of the studied compounds was expressed by IC_50_ values, i.e., by the molar concentration of the compounds causing a 50% decrease in the oxygen evolution rate relative to the untreated control. The comparable IC_50_ value for the selective herbicide 3-(3,4-dichlorophenyl)-1,1-dimethylurea, DCMU (Diuron^®^), was about 0.002 mmol/L. The results are summarized in Table 1.

### 3.3. Statistical Analysis

Statistical analyses were performed using a Statgraphics PlusCenturion XV (Herndon, VA, USA). All measurements were performed in triplicate. Data was expressed as mean ± standard deviation (SD). Analysis of variance (ANOVA) and the least significant difference (LSD) test were applied to determine differences between means. Differences were considered to be significant at *p* ≤ 0.05 confidence level. The one-way analysis of the variance (ANOVA) test was complemented by the Bonferroni’s multicomparison test.

## 4. Conclusions

A series of prepared and characterized eight 1-[(2-chlorophenyl)carbamoyl]naphthalen-2-yl alkylcarbamates **1a**–**1h** and eight 1-[(2-nitrophenyl)carbamoyl]naphthalen-2-yl alkylcarbamates **2a**–**2h** were tested for their activity related to the inhibition of PET in spinach (*Spinacia oleracea* L.) chloroplasts. The highest activity within both series of carbamates was observed for 1-[(2-chlorophenyl)carbamoyl]naphthalen-2-yl propylcarbamate (**1b**, IC_50_ = 80 µM). In spite of the rather low PET-inhibiting activity of the compounds, it was found that they inhibit PET in PS II. Lipophilicity and bulkiness of *N*-alkyl substituent R^2^ seem to be important factors that influence PET-inhibiting activity, as trends for both series are similar. In addition to these parameters, PET-inhibiting activity was also affected by the electronic properties of R^2^ substituent (whereas the influence of PET inhibition on electronic properties for the two series was opposite), and by possible interactions and electron activation of amide and carbamate groups (responsible also for interactions with photosynthetic apparatus) with the spatially close NO_2_ and Cl moieties in the *ortho* position of the anilide ring.

## Figures and Tables

**Table 1:** Structures of the discussed anilides **1**, **2** and carbamates **1a**–**1h**, **2a**–**2h**; predicted clogP values, molar volume MV [cm^−3^], Taft polar constants σ* of R^2^ substituents of compounds and IC_50_ [mmol/L] values related to PET inhibition in spinach chloroplasts of tested compounds in comparison with 3-(3,4-dichlorophenyl)-1,1-dimethylurea (DCMU) standard. IC_50_ values are expressed as mean ± SD (*n* = 3 experiments), the means followed by different letters (a–j) are significantly different at *p* ≤0.05.

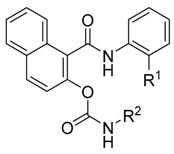						
Comp.	R^1^	R^2^	clogP ^‡^	MV_R2_^‡^ [cm^3^]	σ*_R2_^‡^	PET Inhibition IC_50_ [mmol/L]
**1**	Cl	—	5.03	—	—	0.049 ± 0.002 ^a^
**1a**	Cl	–C_2_H_5_	3.94	47.29	-0.11	0.659 ± 0.046 ^j^
**1b**	Cl	–C_3_H_7_	4.41	63.80	-0.12	0.080 ± 0.002 ^a^
**1c**	Cl	–CH(CH_3_)_2_	4.20	64.18	-0.19	0.271 ± 0.010 ^d^
**1d**	Cl	–C_4_H_9_	4.71	80.31	-0.25	0.589 ± 0.031 ^i^
**1e**	Cl	–C_5_H_11_	5.47	96.81	-0.23	0.396 ± 0.017 ^f^
**1f**	Cl	–C_6_H_13_	6.03	113.32	-0.25	0.358 ± 0.013 ^e^
**1g**	Cl	–C_7_H_15_	6.67	129.83	-0.23	0.263 ± 0.009 ^c,d^
**1h**	Cl	–C_8_H_17_	7.19	146.33	-0.23	0.290 ± 0.010 ^d^
**2**	NO_2_	—	4.45	—	—	0.121 ± 0.004 ^b^
**2a**	NO_2_	–C_2_H_5_	3.58	47.29	-0.11	0.450 ± 0.022 ^g^
**2b**	NO_2_	–C_3_H_7_	3.96	63.80	-0.12	0.365 ± 0.017 ^e,f^
**2c**	NO_2_	–CH(CH_3_)_2_	3.80	64.18	-0.19	0.664 ± 0.041 ^j^
**2d**	NO_2_	–C_4_H_9_	4.32	80.31	-0.25	0.274 ± 0.012 ^d^
**2e**	NO_2_	–C_5_H_11_	5.15	96.81	-0.23	0.233 ± 0.008 ^c^
**2f**	NO_2_	–C_6_H_13_	5.71	113.32	-0.25	0.283 ± 0.012 ^d^
**2g**	NO_2_	–C_7_H_15_	6.81	129.83	-0.23	0.352 ± 0.018 ^e^
**2h**	NO_2_	–C_8_H_17_	7.22	146.33	-0.23	0.487 ± 0.027 ^h^
**DCMU**	—	—	—	—	—	0.002

**^‡^** calculated using ACD/Percepta ver. 2012 (Advanced Chemistry Development, Toronto, ON, Canada).
